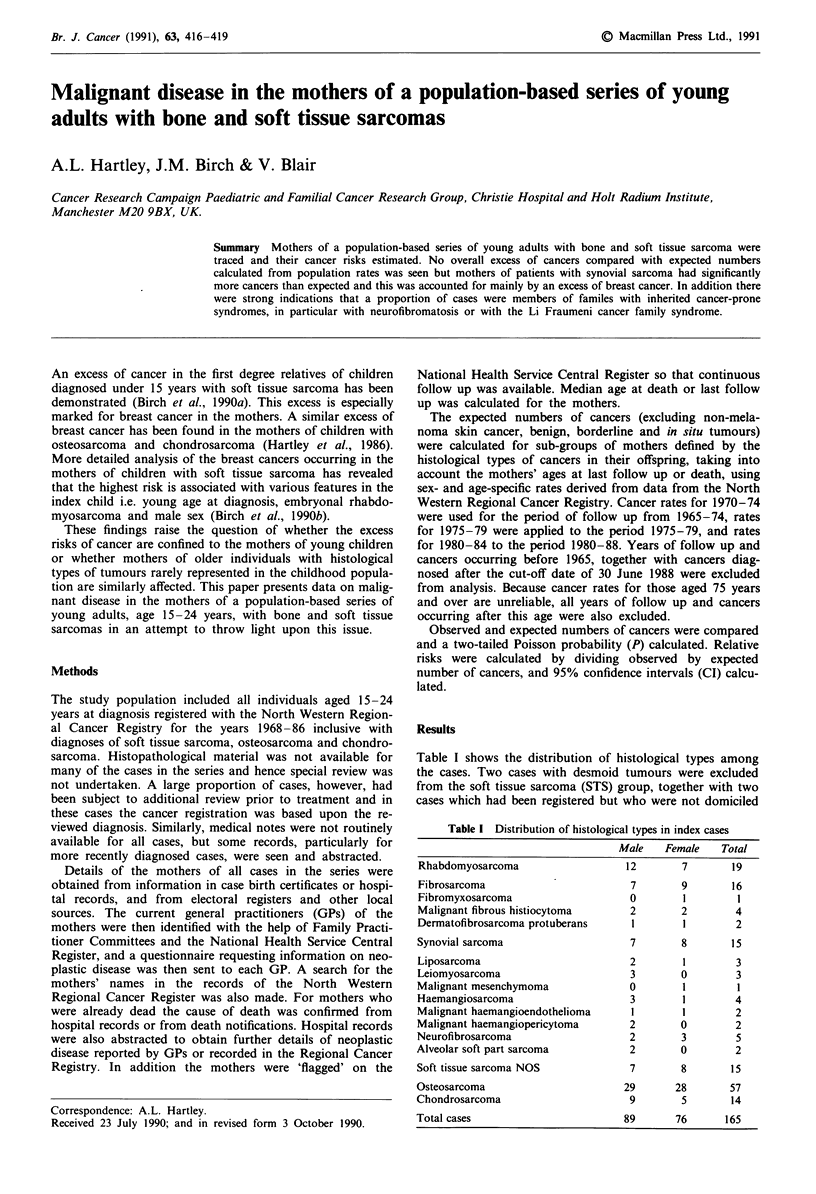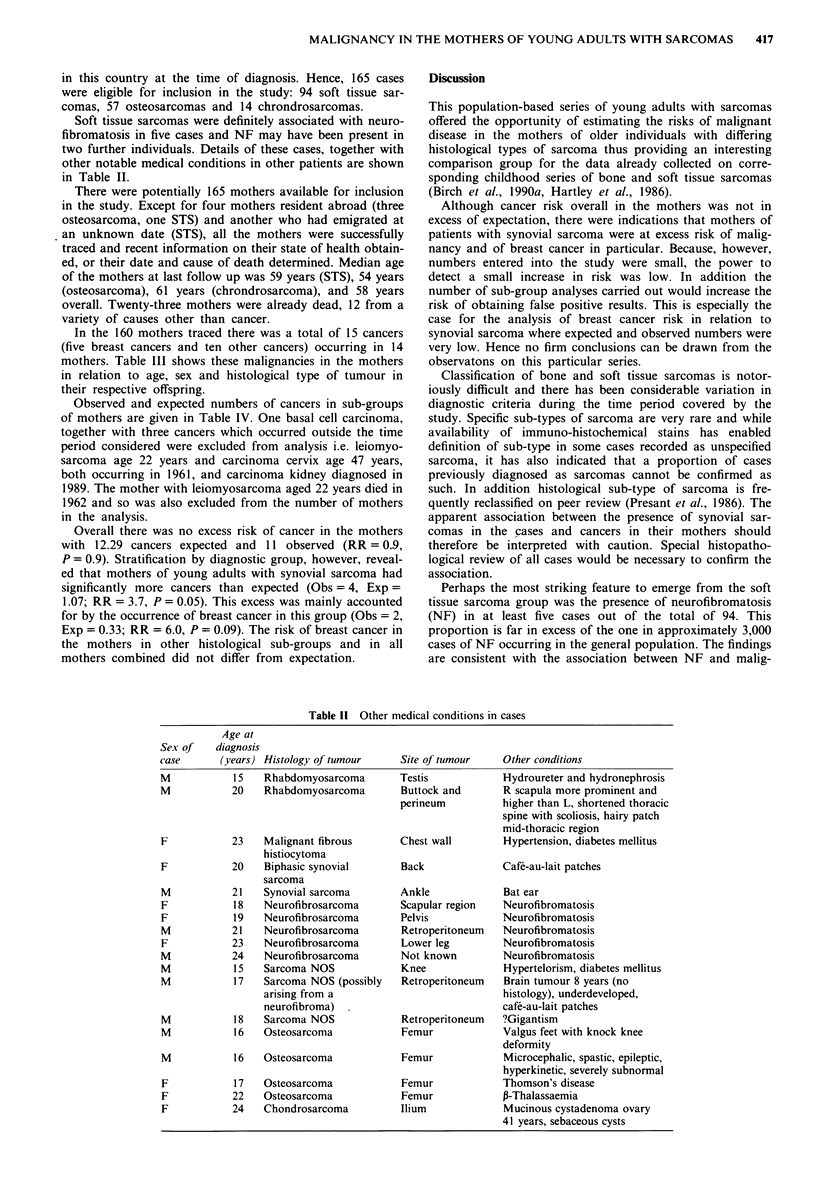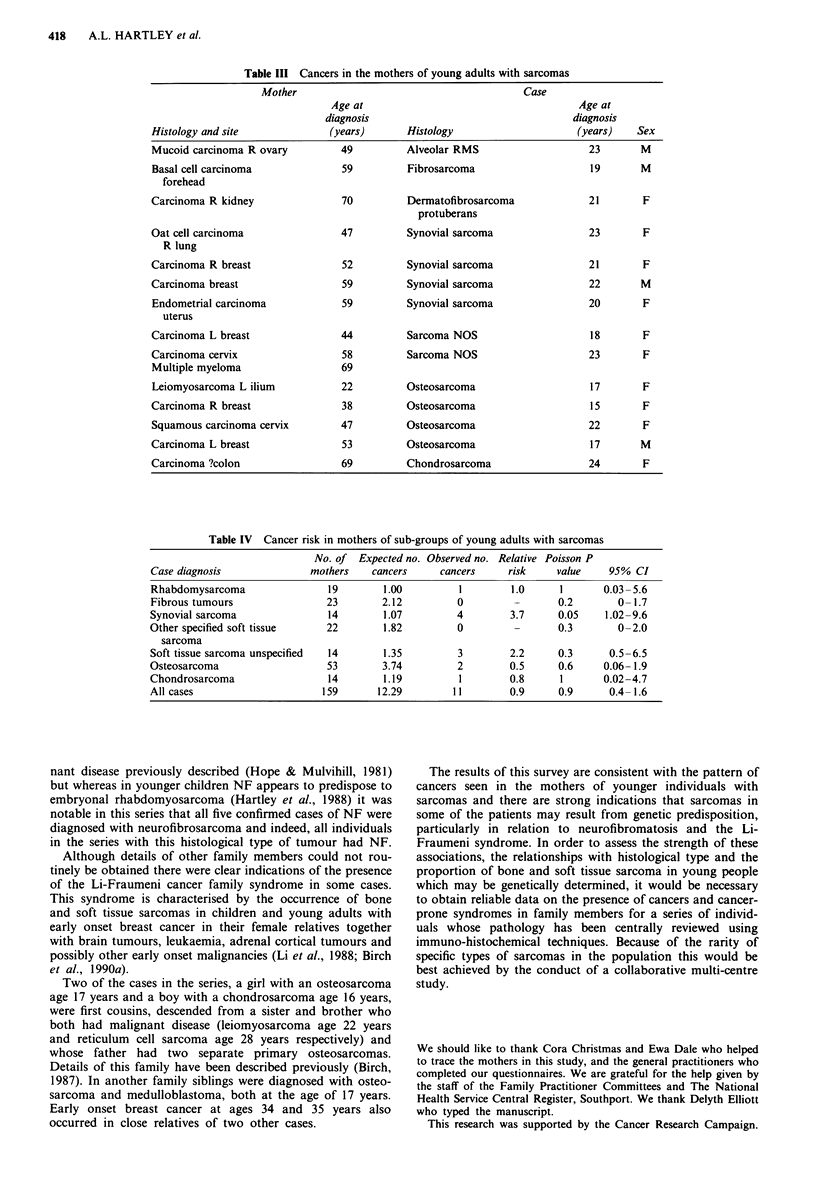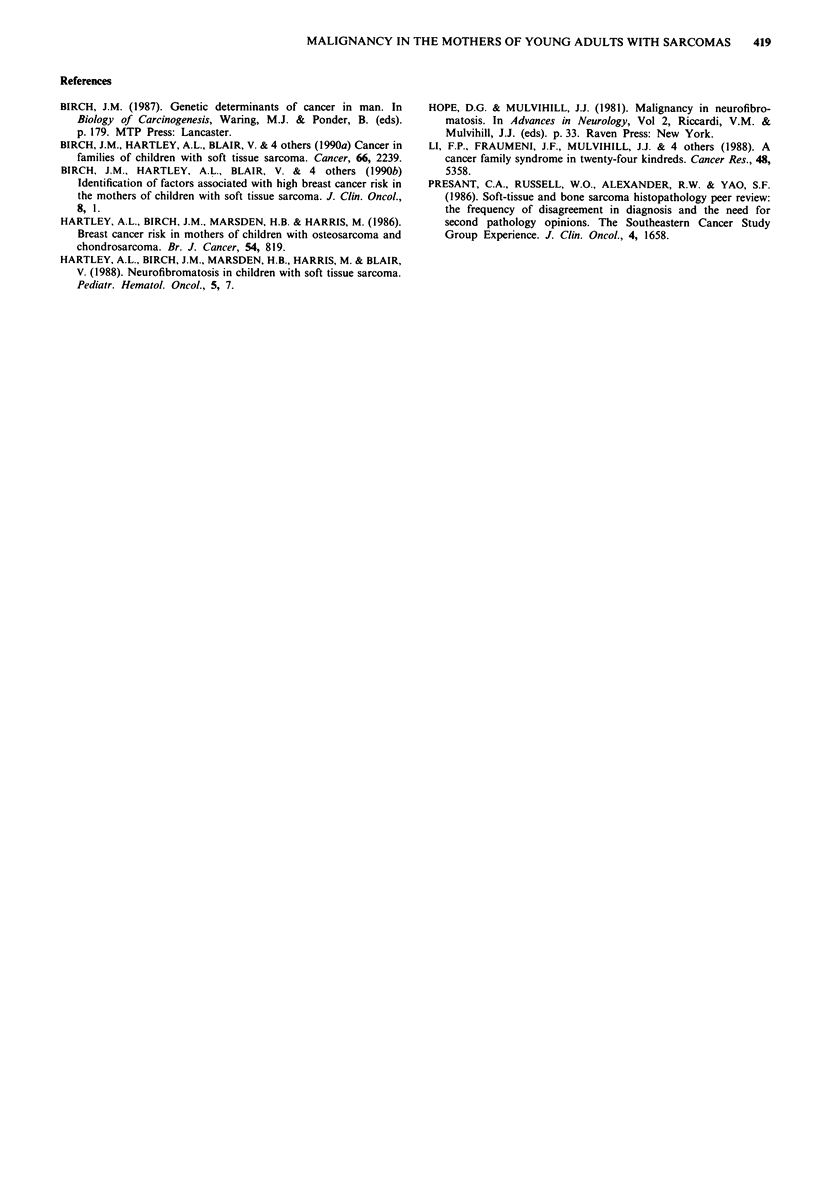# Malignant disease in the mothers of a population-based series of young adults with bone and soft tissue sarcomas.

**DOI:** 10.1038/bjc.1991.96

**Published:** 1991-03

**Authors:** A. L. Hartley, J. M. Birch, V. Blair

**Affiliations:** Cancer Research Campaign, Christie Hospital and Holt Radium Institute, Manchester, UK.

## Abstract

Mothers of a population-based series of young adults with bone and soft tissue sarcoma were traced and their cancer risks estimated. No overall excess of cancers compared with expected numbers calculated from population rates was seen but mothers of patients with synovial sarcoma had significantly more cancers than expected and this was accounted for mainly by an excess of breast cancer. In addition there were strong indications that a proportion of cases were members of families with inherited cancer-prone syndromes, in particular with neurofibromatosis or with the Li Fraumeni cancer family syndrome.


Br. J. Cancer (1991), 63, 416-419 © Macmillan Press Ltd., 1991

### Malignant disease in the mothers of a population-based series of young adults with bone and soft tissue sarcomas

A.L. Hartley, J.M. Birch & V. Blair

*Cancer Research Campaign Paediatric and Familial Cancer Research Group, Christie Hospital and Holt Radium Institute, Manchester M20 9BX, UK.*

**Summary** Mothers of a population-based series of young adults with bone and soft tissue sarcoma were traced and their cancer risks estimated. No overall excess of cancers compared with expected numbers calculated from population rates was seen but mothers of patients with synovial sarcoma had significantly more cancers than expected and this was accounted for mainly by an excess of breast cancer. In addition there were strong indications that a proportion of cases were members of familes with inherited cancer-prone syndromes, in particular with neurofibromatosis or with the Li Fraumeni cancer family syndrome.

An excess of cancer in the first degree relatives of children diagnosed under 15 years with soft tissue sarcoma has been demonstrated (Birch *et al.*, 1990*a*). This excess is especially marked for breast cancer in the mothers. A similar excess of breast cancer has been found in the mothers of children with osteosarcoma and chondrosarcoma (Hartley *et al.*, 1986). More detailed analysis of the breast cancers occurring in the mothers of children with soft tissue sarcoma has revealed that the highest risk is associated with various features in the index child i.e. young age at diagnosis, embryonal rhabdomyosarcoma and male sex (Birch *et al.*, 1990*b*).

These findings raise the question of whether the excess risks of cancer are confined to the mothers of young children or whether mothers of older individuals with histological types of tumours rarely represented in the childhood population are similarly affected. This paper presents data on malignant disease in the mothers of a population-based series of young adults, age 15-24 years, with bone and soft tissue sarcomas in an attempt to throw light upon this issue.

### Methods

The study population included all individuals aged 15-24 years at diagnosis registered with the North Western Regional Cancer Registry for the years 1968-86 inclusive with diagnoses of soft tissue sarcoma, osteosarcoma and chondrosarcoma. Histopathological material was not available for many of the cases in the series and hence special review was not undertaken. A large proportion of cases, however, had been subject to additional review prior to treatment and in these cases the cancer registration was based upon the reviewed diagnosis. Similarly, medical notes were not routinely available for all cases, but some records, particularly for more recently diagnosed cases, were seen and abstracted.

Details of the mothers of all cases in the series were obtained from information in case birth certificates or hospital records, and from electoral registers and other local sources. The current general practitioners (GPs) of the mothers were then identified with the help of Family Practitioner Committees and the National Health Service Central Register, and a questionnaire requesting information on neoplastic disease was then sent to each GP. A search for the mothers' names in the records of the North Western Regional Cancer Register was also made. For mothers who were already dead the cause of death was confirmed from hospital records or from death notifications. Hospital records were also abstracted to obtain further details of neoplastic disease reported by GPs or recorded in the Regional Cancer Registry. In addition the mothers were 'flagged' on the National Health Service Central Register so that continuous follow up was available. Median age at death or last follow up was calculated for the mothers.

The expected numbers of cancers (excluding non-melanoma skin cancer, benign, borderline and *in situ* tumours) were calculated for sub-groups of mothers defined by the histological types of cancers in their offspring, taking into account the mothers' ages at last follow up or death, using sex- and age-specific rates derived from data from the North Western Regional Cancer Registry. Cancer rates for 1970-74 were used for the period of follow up from 1965-74, rates for 1975-79 were applied to the period 1975-79, and rates for 1980-84 to the period 1980-88. Years of follow up and cancers occurring before 1965, together with cancers diagnosed after the cut-off date of 30 June 1988 were excluded from analysis. Because cancer rates for those aged 75 years and over are unreliable, all years of follow up and cancers occurring after this age were also excluded.

Observed and expected numbers of cancers were compared and a two-tailed Poisson probability (*P*) calculated. Relative risks were calculated by dividing observed by expected number of cancers, and 95% confidence intervals (CI) calculated.

### Results

Table I shows the distribution of histological types among the cases. Two cases with desmoid tumours were excluded from the soft tissue sarcoma (STS) group, together with two cases which had been registered but who were not domiciled

**Table I** Distribution of histological types in index cases

| | *Male* | *Female* | *Total* |
|---|---|---|---|
| Rhabdomyosarcoma | 12 | 7 | 19 |
| Fibrosarcoma | 7 | 9 | 16 |
| Fibromyxosarcoma | 0 | 1 | 1 |
| Malignant fibrous histiocytoma | 2 | 2 | 4 |
| Dermatofibrosarcoma protuberans | 1 | 1 | 2 |
| Synovial sarcoma | 7 | 8 | 15 |
| Liposarcoma | 2 | 1 | 3 |
| Leiomyosarcoma | 3 | 0 | 3 |
| Malignant mesenchymoma | 0 | 1 | 1 |
| Haemangiosarcoma | 3 | 1 | 4 |
| Malignant haemangioendothelioma | 1 | 1 | 2 |
| Malignant haemangiopericytoma | 2 | 0 | 2 |
| Neurofibrosarcoma | 2 | 3 | 5 |
| Alveolar soft part sarcoma | 2 | 0 | 2 |
| Soft tissue sarcoma NOS | 7 | 8 | 15 |
| Osteosarcoma | 29 | 28 | 57 |
| Chondrosarcoma | 9 | 5 | 14 |
| Total cases | 89 | 76 | 165 |

Correspondence: A.L. Hartley.
Received 23 July 1990; and in revised form 3 October 1990.

in this country at the time of diagnosis. Hence, 165 cases were eligible for inclusion in the study: 94 soft tissue sarcomas, 57 osteosarcomas and 14 chrondrosarcomas.

Soft tissue sarcomas were definitely associated with neurofibromatosis in five cases and NF may have been present in two further individuals. Details of these cases, together with other notable medical conditions in other patients are shown in Table II.

There were potentially 165 mothers available for inclusion in the study. Except for four mothers resident abroad (three osteosarcoma, one STS) and another who had emigrated at an unknown date (STS), all the mothers were successfully traced and recent information on their state of health obtained, or their date and cause of death determined. Median age of the mothers at last follow up was 59 years (STS), 54 years (osteosarcoma), 61 years (chrondrosarcoma), and 58 years overall. Twenty-three mothers were already dead, 12 from a variety of causes other than cancer.

In the 160 mothers traced there was a total of 15 cancers (five breast cancers and ten other cancers) occurring in 14 mothers. Table III shows these malignancies in the mothers in relation to age, sex and histological type of tumour in their respective offspring.

Observed and expected numbers of cancers in sub-groups of mothers are given in Table IV. One basal cell carcinoma, together with three cancers which occurred outside the time period considered were excluded from analysis i.e. leiomyosarcoma age 22 years and carcinoma cervix age 47 years, both occurring in 1961, and carcinoma kidney diagnosed in 1989. The mother with leiomyosarcoma aged 22 years died in 1962 and so was also excluded from the number of mothers in the analysis.

Overall there was no excess risk of cancer in the mothers with 12.29 cancers expected and 11 observed (RR = 0.9, $P = 0.9$). Stratification by diagnostic group, however, revealed that mothers of young adults with synovial sarcoma had significantly more cancers than expected (Obs = 4, Exp = 1.07; RR = 3.7, $P = 0.05$). This excess was mainly accounted for by the occurrence of breast cancer in this group (Obs = 2, Exp = 0.33; RR = 6.0, $P = 0.09$). The risk of breast cancer in the mothers in other histological sub-groups and in all mothers combined did not differ from expectation.

### Discussion

This population-based series of young adults with sarcomas offered the opportunity of estimating the risks of malignant disease in the mothers of older individuals with differing histological types of sarcoma thus providing an interesting comparison group for the data already collected on corresponding childhood series of bone and soft tissue sarcomas (Birch *et al.*, 1990*a*, Hartley *et al.*, 1986).

Although cancer risk overall in the mothers was not in excess of expectation, there were indications that mothers of patients with synovial sarcoma were at excess risk of malignancy and of breast cancer in particular. Because, however, numbers entered into the study were small, the power to detect a small increase in risk was low. In addition the number of sub-group analyses carried out would increase the risk of obtaining false positive results. This is especially the case for the analysis of breast cancer risk in relation to synovial sarcoma where expected and observed numbers were very low. Hence no firm conclusions can be drawn from the observatons on this particular series.

Classification of bone and soft tissue sarcomas is notoriously difficult and there has been considerable variation in diagnostic criteria during the time period covered by the study. Specific sub-types of sarcoma are very rare and while availability of immuno-histochemical stains has enabled definition of sub-type in some cases recorded as unspecified sarcoma, it has also indicated that a proportion of cases previously diagnosed as sarcomas cannot be confirmed as such. In addition histological sub-type of sarcoma is frequently reclassified on peer review (Presant *et al.*, 1986). The apparent association between the presence of synovial sarcomas in the cases and cancers in their mothers should therefore be interpreted with caution. Special histopathological review of all cases would be necessary to confirm the association.

Perhaps the most striking feature to emerge from the soft tissue sarcoma group was the presence of neurofibromatosis (NF) in at least five cases out of the total of 94. This proportion is far in excess of the one in approximately 3,000 cases of NF occurring in the general population. The findings are consistent with the association between NF and malig-

**Table II** Other medical conditions in cases

| *Sex of case* | *Age at diagnosis (years)* | *Histology of tumour* | *Site of tumour* | *Other conditions* |
|---|---|---|---|---|
| M | 15 | Rhabdomyosarcoma | Testis | Hydroureter and hydronephrosis |
| M | 20 | Rhabdomyosarcoma | Buttock and perineum | R scapula more prominent and higher than L, shortened thoracic spine with scoliosis, hairy patch mid-thoracic region |
| F | 23 | Malignant fibrous histiocytoma | Chest wall | Hypertension, diabetes mellitus |
| F | 20 | Biphasic synovial sarcoma | Back | Café-au-lait patches |
| M | 21 | Synovial sarcoma | Ankle | Bat ear |
| F | 18 | Neurofibrosarcoma | Scapular region | Neurofibromatosis |
| F | 19 | Neurofibrosarcoma | Pelvis | Neurofibromatosis |
| M | 21 | Neurofibrosarcoma | Retroperitoneum | Neurofibromatosis |
| F | 23 | Neurofibrosarcoma | Lower leg | Neurofibromatosis |
| M | 24 | Neurofibrosarcoma | Not known | Neurofibromatosis |
| M | 15 | Sarcoma NOS | Knee | Hypertelorism, diabetes mellitus |
| M | 17 | Sarcoma NOS (possibly arising from a neurofibroma) | Retroperitoneum | Brain tumour 8 years (no histology), underdeveloped, café-au-lait patches |
| M | 18 | Sarcoma NOS | Retroperitoneum | ?Gigantism |
| M | 16 | Osteosarcoma | Femur | Valgus feet with knock knee deformity |
| M | 16 | Osteosarcoma | Femur | Microcephalic, spastic, epileptic, hyperkinetic, severely subnormal |
| F | 17 | Osteosarcoma | Femur | Thomson's disease |
| F | 22 | Osteosarcoma | Femur | β-Thalassaemia |
| F | 24 | Chondrosarcoma | Ilium | Mucinous cystadenoma ovary 41 years, sebaceous cysts |

**Table III** Cancers in the mothers of young adults with sarcomas

| *Mother* Histology and site | Age at diagnosis (years) | *Case* Histology | Age at diagnosis (years) | Sex |
|---|---|---|---|---|
| Mucoid carcinoma R ovary | 49 | Alveolar RMS | 23 | M |
| Basal cell carcinoma forehead | 59 | Fibrosarcoma | 19 | M |
| Carcinoma R kidney | 70 | Dermatofibrosarcoma protuberans | 21 | F |
| Oat cell carcinoma R lung | 47 | Synovial sarcoma | 23 | F |
| Carcinoma R breast | 52 | Synovial sarcoma | 21 | F |
| Carcinoma breast | 59 | Synovial sarcoma | 22 | M |
| Endometrial carcinoma uterus | 59 | Synovial sarcoma | 20 | F |
| Carcinoma L breast | 44 | Sarcoma NOS | 18 | F |
| Carcinoma cervix<br>Multiple myeloma | 58<br>69 | Sarcoma NOS | 23 | F |
| Leiomyosarcoma L ilium | 22 | Osteosarcoma | 17 | F |
| Carcinoma R breast | 38 | Osteosarcoma | 15 | F |
| Squamous carcinoma cervix | 47 | Osteosarcoma | 22 | F |
| Carcinoma L breast | 53 | Osteosarcoma | 17 | M |
| Carcinoma ?colon | 69 | Chondrosarcoma | 24 | F |

**Table IV** Cancer risk in mothers of sub-groups of young adults with sarcomas

| Case diagnosis | No. of mothers | Expected no. cancers | Observed no. cancers | Relative risk | Poisson P value | 95% CI |
|---|---|---|---|---|---|---|
| Rhabdomysarcoma | 19 | 1.00 | 1 | 1.0 | 1 | 0.03–5.6 |
| Fibrous tumours | 23 | 2.12 | 0 | – | 0.2 | 0–1.7 |
| Synovial sarcoma | 14 | 1.07 | 4 | 3.7 | 0.05 | 1.02–9.6 |
| Other specified soft tissue sarcoma | 22 | 1.82 | 0 | – | 0.3 | 0–2.0 |
| Soft tissue sarcoma unspecified | 14 | 1.35 | 3 | 2.2 | 0.3 | 0.5–6.5 |
| Osteosarcoma | 53 | 3.74 | 2 | 0.5 | 0.6 | 0.06–1.9 |
| Chondrosarcoma | 14 | 1.19 | 1 | 0.8 | 1 | 0.02–4.7 |
| All cases | 159 | 12.29 | 11 | 0.9 | 0.9 | 0.4–1.6 |

nant disease previously described (Hope & Mulvihill, 1981) but whereas in younger children NF appears to predispose to embryonal rhabdomyosarcoma (Hartley *et al.*, 1988) it was notable in this series that all five confirmed cases of NF were diagnosed with neurofibrosarcoma and indeed, all individuals in the series with this histological type of tumour had NF.

Although details of other family members could not routinely be obtained there were clear indications of the presence of the Li-Fraumeni cancer family syndrome in some cases. This syndrome is characterised by the occurrence of bone and soft tissue sarcomas in children and young adults with early onset breast cancer in their female relatives together with brain tumours, leukaemia, adrenal cortical tumours and possibly other early onset malignancies (Li *et al.*, 1988; Birch *et al.*, 1990*a*).

Two of the cases in the series, a girl with an osteosarcoma age 17 years and a boy with a chondrosarcoma age 16 years, were first cousins, descended from a sister and brother who both had malignant disease (leiomyosarcoma age 22 years and reticulum cell sarcoma age 28 years respectively) and whose father had two separate primary osteosarcomas. Details of this family have been described previously (Birch, 1987). In another family siblings were diagnosed with osteosarcoma and medulloblastoma, both at the age of 17 years. Early onset breast cancer at ages 34 and 35 years also occurred in close relatives of two other cases.

The results of this survey are consistent with the pattern of cancers seen in the mothers of younger individuals with sarcomas and there are strong indications that sarcomas in some of the patients may result from genetic predisposition, particularly in relation to neurofibromatosis and the Li-Fraumeni syndrome. In order to assess the strength of these associations, the relationships with histological type and the proportion of bone and soft tissue sarcoma in young people which may be genetically determined, it would be necessary to obtain reliable data on the presence of cancers and cancer-prone syndromes in family members for a series of individuals whose pathology has been centrally reviewed using immuno-histochemical techniques. Because of the rarity of specific types of sarcomas in the population this would be best achieved by the conduct of a collaborative multi-centre study.

We should like to thank Cora Christmas and Ewa Dale who helped to trace the mothers in this study, and the general practitioners who completed our questionnaires. We are grateful for the help given by the staff of the Family Practitioner Committees and The National Health Service Central Register, Southport. We thank Delyth Elliott who typed the manuscript.

This research was supported by the Cancer Research Campaign.

### References

BIRCH, J.M. (1987). Genetic determinants of cancer in man. In *Biology of Carcinogenesis*, Waring, M.J. & Ponder, B. (eds). p. 179. MTP Press: Lancaster.

BIRCH, J.M., HARTLEY, A.L., BLAIR, V. & 4 others (1990*a*) Cancer in families of children with soft tissue sarcoma. *Cancer*, **66**, 2239.

BIRCH, J.M., HARTLEY, A.L., BLAIR, V. & 4 others (1990*b*) Identification of factors associated with high breast cancer risk in the mothers of children with soft tissue sarcoma. *J. Clin. Oncol.*, **8**, 1.

HARTLEY, A.L., BIRCH, J.M., MARSDEN, H.B. & HARRIS, M. (1986). Breast cancer risk in mothers of children with osteosarcoma and chondrosarcoma. *Br. J. Cancer*, **54**, 819.

HARTLEY, A.L., BIRCH, J.M., MARSDEN, H.B., HARRIS, M. & BLAIR, V. (1988). Neurofibromatosis in children with soft tissue sarcoma. *Pediatr. Hematol. Oncol.*, **5**, 7.

HOPE, D.G. & MULVIHILL, J.J. (1981). Malignancy in neurofibromatosis. In *Advances in Neurology*, Vol 2, Riccardi, V.M. & Mulvihill, J.J. (eds). p. 33. Raven Press: New York.

LI, F.P., FRAUMENI, J.F., MULVIHILL, J.J. & 4 others (1988). A cancer family syndrome in twenty-four kindreds. *Cancer Res.*, **48**, 5358.

PRESANT, C.A., RUSSELL, W.O., ALEXANDER, R.W. & YAO, S.F. (1986). Soft-tissue and bone sarcoma histopathology peer review: the frequency of disagreement in diagnosis and the need for second pathology opinions. The Southeastern Cancer Study Group Experience. *J. Clin. Oncol.*, **4**, 1658.